# Risk communication and human biomonitoring: which practical lessons from the Belgian experience are of use for the EU perspective?

**DOI:** 10.1186/1476-069X-7-S1-S11

**Published:** 2008-06-05

**Authors:** Hans Keune, Bert Morrens, Ilse Loots

**Affiliations:** 1 Faculty of Political and Social Sciences, University of Antwerp, Sint Jacobstraat 2, B – 2000 Antwerpen, Belgium

## Abstract

**Background:**

In order to investigate and monitor environmental health in Flanders (the Dutch speaking part of Belgium), the Flemish government funded the Centre of Expertise for Environment and Health, which started a human biomonitoring campaign in 2001. In addition to environmental health experts measuring environmental pollutants and health effects in human beings, social scientific experts at the Centre focus on risk communication associated with the human biomonitoring campaign.

**Methods:**

In the literature about risk communication an evolution can be traced from traditional, one-way communication, restricted to the dissemination of information from experts to the public, to more modern, two-way risk communication, with a focus on participation and cooperation between scientists, policy-makers and the public.

Within the Centre of Expertise for Environment and Health this discourse was first translated into some general principles and guidelines for external communication, at a 'Ten Commandments level'. These principles needed to be incorporated in the day-to-day practice of human biomonitoring research.

**Results:**

The social scientific experts at the Centre developed a combined risk communication strategy. On the one hand the strategy consists of traditional risk communication for external communication purposes, for example information meetings and digital newsletters. On the other hand it consists of a step by step approach of incorporating more modern risk communication, for example a risk perception questionnaire, dialogical experiments for involving local stakeholders, and an action-plan for interpreting results for policy making.

**Conclusion:**

With a parallel strategy of traditional and modern communication, of external and internal reflection, and through different social scientific projects, the Flemish Centre of Expertise of Environment and Health incorporates risk communication in the day-to-day practice of human biomonitoring research. A direct and continuous involvement of the social scientist, an openness between all colleagues involved, and the awareness of a fine balance between quality and practicability are important success factors. These lessons may be helpful and inspirational for a European human biomonitoring project.

## Background

In 2001 twelve Centres for Policy Relevant Research were initiated in Flanders (the Dutch speaking part of Belgium) by the Flemish government. Their main task is to carry out scientific research on priority issues for government policy. A steering group consisting of representatives of governmental institutions is attached to each Centre. In the steering group, policy makers not only follow up on research outcomes, but also discuss the knowledge production and valorisation with the researchers.

One of these twelve Centres is the Centre of Expertise for Environment and Health [1], which started at the end of 2001 to be run for a period of five years. At this Centre, environmental health experts from all Flemish universities, the Dutch University of Maastricht, and two research institutes jointly investigate the complex relationship between environment and health. In addition the Centre houses a social scientific expert unit which focuses on risk communication, risk perception, and on processes of knowledge production, interpretation, deliberation and cooperation between different scientific disciplines and other social actors. After the initial programme period, the decision was made to continue the Centre for a further five years (2007–2011), including the social scientific unit. This decision seemed to recognise not only the policy relevance of environmental health monitoring and research, but also the importance of its societal dimension.

The main task of the Centre of Expertise for Environment and Health is the human biomonitoring project, which investigates the very complex relation between environmental pollution and human health. This is done by measuring both a number of selected pollutants and certain health effects in human beings, focusing on three different target groups: newborn babies, adolescents and adults. Each campaign is carried out in eight areas of Flanders (200 participants per area and age group). These areas have different environmental characteristics, such as industrialized, rural (the countryside), urbanized, near waste incinerators, and near fruit orchards. Part of the objective of this human biomonitoring is to focus on a comparison of exposure and health effects associated with these different types of area specific environmental pressure.

In this paper we will focus on the risk communication activities over the past five years and list lessons learned that could potentially be of use for the perspective of biomonitoring within the European Union.

## Methods

### Risk communication

#### A brief history of risk communication

Risk communication occurs whenever there is an exchange of information among interested parties about the nature, magnitude, significance or control of a risk. Information about risks can be communicated through a variety of channels, ranging from media reports and warning labels to public meetings or hearings [2]. However, risk communication has long been dominated by 'top-down' technocratic approaches that can be characterised as expert to public monologues. Such approaches contrast strongly with the more 'open' approaches based on concepts such as partnership and dialogue that have recently moved to centre stage in risk communication.

Government, science and industry long shared the belief that communication about risks was unnecessary as long as those risks were controllable and kept at acceptable levels. Risk experts needed to focus on risk control by using quantitative risk analyses their job being completed once the numbers came out satisfactorily. However, risk control appeared far from easy, as risks were no longer unwanted side-effects of production, but an inherent characteristic of our modern industrial risk society [3]. Furthermore a significant gap was observed between the risk perception of the experts practising risk assessment on the one hand, and the public on the other hand [4].

Facing more and more public resistance to technological projects and risk-based decision-making in the 1970's and 80's, technicians and experts tended to present their own views as objective and rational assessments of the real risks, whereas the views of laypeople were presented as a false understanding of reality resulting from a subjective, intuitive, emotional and irrational perception. The public therefore needed to be 'educated', in order to fill the observed 'perception gap', by emphasizing the conveyance of technical information from experts to laypeople [5]. Scientists, policy makers and industry tried to persuade the public of the veracity of the expert point of view on risks through presenting scientific or technical information at public meetings, and in reports and other communication messages. Risk communication in this sense is traditional, one-way communication where information is channelled from experts to a general audience, and where the former tries to inform the latter about "the truth".

But such risk communication faced severe difficulties since there was a growing lack of trust and credibility in science and policy amongst the public [2,6]. According to Kasperson et al. [7] "a broad-based loss of trust in the leaders of major social institutions and in the institutions themselves has occurred over the past three decades". Especially after some major technical disasters in 1986 (e.g. the Chernobyl catastrophe and the space shuttle Challenger accident) there was what Renn [8] calls a 'massive mood of non-acceptance' towards the chemical industry, waste recycling plants, road building schemes, airport expansions, etc.

It became clear that risk communication restricted to conveying technical information will ultimately fail to communicate because it ignores the historic and social context of risks [5]. Social constructivism and cultural theory declare that risks have different meanings for different people, that they are socially constructed [9]. Consequently, risk became the domain of social scientists, and not just of technical experts. Risk communication should thus consider two forms of rationality, technical and social. Expert and lay opinions need to be perceived as complementing rather than competing with each other. The most effective way to combine these different perspectives is by involving the public in the communication process, by making them partners.

Participatory and dialogical processes are thus needed in risk communication to combine technical expertise, rational decision making, and public values and preferences. This participatory approach has attracted increasing attention, and is, for example, being supported by TRUSTNET, the interdisciplinary European Union network involved in the field of risk governance. The participatory human biomonitoring in Belgian Flanders described in this paper is one of the nine innovative processes making up this network. In a final report TRUSTNET stated that "(...) a pragmatic methodology for cooperative inquiries is needed to address complex issues (in particular risk issues) impacting multiple aspects of people's actual life. This methodology involves citizens, civil society organizations and other stakeholders (local communities, interest groups, etc), working together with an interdisciplinary group of scientists and experts through processes of cooperative inquiry, to investigate a problem which matters to the public" [10].

#### Benefits of modern risk communication

One of the main problems in traditional risk communication is ignoring the fact that different perceptions and perspectives are relevant and should be respected. Overcoming the gaps between science, policy and the public is still one of the biggest challenges of modern risk communication. Mutual understanding and participation are necessary to create trust in order to solve problems that are both scientifically and socially complex.

There are three main goals for involving public participation in decision-making processes and policy relevant research [11,12]. First, the value of a final decision is higher when non-scientific (e.g. local) expert knowledge is included, since science itself suffers from many uncertainties and unknowns, especially in the complex relationship between environment and health. Second, the legitimacy of the final outcome is higher when potentially affected parties can state their own case before their peers and have an equal chance to influence the outcome. Participation is therefore likely to increase public support for the policy decision-making process. Third, it is a way of implementing democracy. Public participation is identified with the way democratic government should conduct itself in public decision-making activities.

However, risk communication is no panacea, and avoiding all conflicts is not a realistic, nor a legitimate, goal for risk communication, as Fischhoff rightly stated. "The best-case scenario for risk communication (and risk management) is having fewer, but better conflicts" [13].

#### Risk communication and human biomonitoring: rules of the game

During the first year of the Centre of Expertise for Environment and Health, we worked on guidelines for the external communication of the Centre [14]. This was done in close cooperation with other actors within the Centre, plus researchers and government representatives. These guidelines are intended to involve all relevant persons or organisations (scientists, experts, policy makers, citizens and interest groups) in the work of the Centre.

The main written down general principles are that:

• Environmental and health problems are looked at differently depending on differences in personal background. Differences in risk perceptions are based on differences in the problem definition.

• All forms of knowledge (science, intuition, experience, values) are relevant and should be taken seriously.

• As a consequence of the complex character of environmental – and health research, scientific controversies and uncertainties are inevitable.

These communication principles were introduced by the social scientists; gradually policy makers and exact scientists also familiarized themselves with these principles.

Some practical directives for the communication of human biomonitoring research results are also written down:

• Transparency: the Centre wants to be transparent about its work, not only concerning outcomes or interpretations, but also in relation to choices made during the process of design of the study, research, interpretation procedures, policy options, etc.

• Participants first: participants in the research are informed first about research results, before the press and the general public. Note that this concerns results on a general level, individual results being provided only to the participants.

On the issue of transparency, attention should be drawn to the so-called 'Aarhus Convention' [15], which establishes a number of rights for the public (individuals and their associations) with regard to the environment:

• The right of everyone to receive environmental information that is held by public authorities ("access to environmental information").

• The right to participate in environmental decision-making ("public participation in environmental decision-making").

• The right to review procedures to challenge public decisions that have been made without respecting the two aforementioned rights or environmental law in general ("access to justice").

This Convention was approved by the European Union in 2005 [16].

#### Communication in practice: from a 'Ten Commandments level' to practical strategy

It is relatively easy for a politician to say he or she is truly democratic. However, in day-to-day practice, democracy is very complex and has no objective ideal or perfect practical form. This is also true for modern risk communication. At the 'Ten Commandments level' the main principles for external risk communication of the Centre of Expertise for Environment and Health are relatively easily understood and accorded by the actors involved. In practice, though, many of the practical problems and complexities are often underestimated. Practice will thus constitute a litmus-test for consensus at a 'Ten Commandments level': differences in vision, interpretation and preference will swiftly become apparent. Furthermore, we have to take into account the sometimes rather large discrepancy between the social scientific perspective on issues like risk communication and the way other scientific disciplines or policy representatives perceive it.

The strategy we developed for the implementation of the risk communication principles in practice tried to take into account the issues described here, resulting in a step by step approach with parallel processes of communication, both external and internal within the Centre. Before implementing modern approaches to communication on research and policy for the sake of openness and the involvement of societal actors however, it is important first to think carefully, internally, backstage [17] about what one hopes to achieve with the communication. To keep both the doors and windows of the Centre closed to the outside world until the wise men and women of the Centre have made up their minds for example would be unwise. Since the Centre was introduced in Flanders and started human biomonitoring research in several regions, the Centre was no longer anonymous and not to communicate would be to contradict the guidelines for external communication of the Centre. However, to make the giant leap to adopting modern risk communication was simply not possible. We therefore opted for a combined strategy of traditional and modern risk communication, in close discussion and cooperation with the other disciplines and policy actors.

#### The mixed methods approach

This approach then posed the methodological question of how to incorporate principles from modern risk communication into the day-to-day practice of human biomonitoring research. As environmental health risks involve a mixture of complexity, uncertainty and ambiguity, investigating these risks and communicating about them demands what Klinke and Renn [18] call 'a multidisciplinary approach using mixed methods'. Compared to single approach designs, mixed methods research can answer questions in a better way, allows stronger inferences, and provides opportunities for presenting a wider range of divergent views [19]. Quantitative methods provide relatively standardized, efficient, amenable information, which can be easily summarized and analyzed. Qualitative methods add contextual and cultural dimensions, which deepen the study by providing more natural information. Combining these two can thus be considered a 'third approach' [20], transcending the separatism between quantitative and qualitative research. When using such mixed methods approach one needs to adopt a transactional and subjectivist epistemology [21], a framework where knowledge is by definition considered plural and uncertain. This contrasts with the more positivist assumption that knowledge is hard, real and capable of being transmitted in tangible form. Pragmatism has increasingly become the philosophical rationale for mixed methods practice [20]. The bottom line is that research approaches should be mixed in ways that offer the best opportunities for answering complex research questions.

As social scientific experts at the Centre of Expertise for Environment and Health share these assumptions, the risk communication strategy was implemented in the human biomonitoring project using a mixture of different methods and approaches. Exactly how this was done is the subject of the next paragraph.

## Results

As described above, at the start of the Centre for Environment and Health we developed a parallel communication strategy of traditional (one-way) risk communication for external communication purposes and an internal reflection on more modern (two-way) risk communication within the Centre. Even though the up-to-date knowledge and experience of social scientific approaches of modern risk communication was welcomed and accorded by the medical and environmental scientific colleagues and was asked for by policy makers at the beginning of the Centre, the implementation in practice appeared to be far less obvious. Clearly this rather unknown territory (for the non social scientific experts) needed to be explored in cooperation and deliberation with our colleagues as well as the representatives from policy.

**Figure 1:**
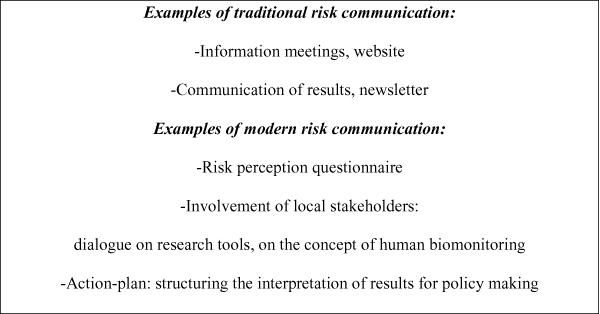
Examples of risk communication activities within the Flemish Centre of Expertise of Environment and Health.

First we will describe the way we implemented traditional risk communication (from experts to the public) from the beginning. Second we will describe developments with regard to modern risk communication; an overview in Figure 1.

### Traditional from the start

As mentioned above, we started with traditional risk communication right from the inception of the Centre of Expertise for Environment and Health, the main characteristic being (mainly) one-sided information from the Centre to society. We will introduce two aspects: communication about the human biomonitoring research and communication tools.

In order to be transparent about the research, the Centre and policy makers organized information meetings before the actual start of the research in order to introduce the aims and means of the research in different regions in Flanders. To further the dissemination of information a website was also set up which was gradually improved over time both in appearance and content.

In order to be transparent about the research results, research reports were made public. We also organized, together with policy representatives, press conferences and information meetings. Part of the complexity of communication of results at the general level was timing, leading to lot of discussion about how best to combine for example a press conference with a minister, an information meeting, informing local authorities and the principle of 'participants first'. While individual participants should by rights receive the reports (non-technical versions) before the press, at the same time the risk of participants leaking information to the press before a ministerial press conference should be taken into account. Very precise timing and tight schedules were therefore essential.

With regard to the individual results participants were given the opportunity to choose between several options:

• To receive the individual results at home

• To have the individual results sent to their general practitioner

• To receive no individual results at all

Communication of individual results is complicated by the fact that interpretation on an individual level is often difficult. In order to give scientific interpretation that meaningful for individuals, the quality of the biomarkers used in the human biomonitoring research is essential, and not all markers are good predictors for health risks on an individual level. Another essential factor is the availability of reference values or norms with regard to health risks. Only with regard to lead (international) norms are available. This means that individual results have to be treated very carefully. The Centre provided only the international scientific information available, together with information about uncertainties or unknowns. Information was also given on the possible risks of certain substances and any means which were available to lower the risks. The network of health and environmental experts working together with the Centre were informed about this, as well as general practitioners (if requested by participants).

**Figure 2:**
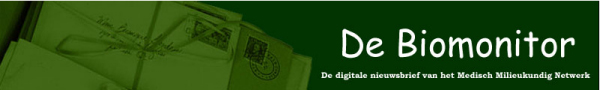
The digital newspaper called the '*Biomonitor*'.

At a later stage also a digital newsletter was developed: 'De Biomonitor' (the Biomonitor, figure 2), whose editorial board is made up of both representatives from the administration (Health and Environment), the network of health and environmental experts working together with the Centre and scientists at the Centre. Part of the aim of the newsletter is to publish the research results from the human biomonitoring research or related subjects. We also invite 'outsiders' to give their opinions about the research. Furthermore in principle everyone is free to submit an article or a comment.

### Step by step experimental modern risk communication

In order to make social scientific concepts of modern risk communication operational, a cooperative step by step approach was developed. Three elements are important here:

• Practicality: the rather tight time schedules applied to the human biomonitoring research limit the options available to fulfil all our communication ambitions.

• Cooperation and reflective learning: we need support from non social scientific colleagues and representatives from policy, in order to make such activities worthwhile and to potentially integrate such activities into the routine of the work at the Centre. We hope to make the actors involved more aware of modern risk communication and help articulate their own preferences.

• Risk communication qualities: the quality of our work should be guaranteed to some extent in order to take responsibility for it as experts in this field.

We needed to find a balance between these three elements.

In the very beginning our strategy was limited to proposing experimental projects with the following characteristics:

• Small: preferably small scale initiatives, requiring limited effort and relatively easy to implement

• Safe: experiments free from obligations to the Centre or policy makers for example with regard to the actual use of input from stakeholders or the public.

• No (extra) dead weight for the human biomonitoring train: the tight time schedule had to be respected. The research train was already on the move and could not be put on hold for the sake of for example extensive discussions on research design.

• Relevance to the human biomonitoring effort from a risk communication perspective: these experiments should open up options for more two-way risk communication on human biomonitoring related issues.

• Relevance to the aspect of policy relevant research: both the biomonitoring and the risk communication activities should be relevant for the policy uptake of issues of environment and health.

We will briefly introduce some of these experiments: a risk perception questionnaire, two experiments involving local stakeholders, and an Action-plan for interpreting results for policy making.

### Risk perception questionnaire

Differences in risk perception are related to a diversity of factors. In addition to scientific factors, social factors also have a significant impact [22-24] e.g. whether people are voluntarily exposed to risks, and the distribution of costs and benefits of risk-generating activities such as industry. Of equal importance is the level of trust people have in individuals or organisations that are responsible for risk management [6,25].

Risk perception research is useful for several reasons. One obvious reason is that purely technical or quantitative research methods cannot explain why people perceive risks as they do, and technical or quantitative research methods are limited because of blind spots. Moreover, understanding risk perception is valuable for risk management [23]. As such an important tool in risk management, risk communication needs to take into account risk perception; one of the main problems in risk communication is ignoring the fact that different perceptions are relevant and should be respected. In order to tackle complex problems such as environmental health problems, it is necessary to incorporate different forms of knowledge of these problems as well as respect the fact that professionals and non-professionals may perceive problems quite differently.

#### Opportunity in relation to human biomonitoring

To measure the exposure to environmental pollutants and health effects in human beings, biomarkers are used. Exposure markers measure the amount of chemicals present in tissue fluids such as blood. Markers for effect measure the possible biological and health impacts of these exposures. Also other factors may influence these markers, such as lifestyle, health status, working conditions, and food intake. If we want to investigate the contribution of environmental exposure to the biomarkers, we need to correct for the contribution of other factors, the confounding factors and co-variables. Many of these co-variables are quantified through questionnaires. Social scientists at the Centre of Expertise for Environment and Health suggested using this opportunity to add a short questionnaire on perception with regard to environmental risks for human health [26].

#### Questionnaire

The risk perception questionnaire touched upon the following topics:

• Do respondents experience environmental problems in their neighbourhood? And do they believe these problems create health risks for them?

• To what extent do respondents trust actors involved with environmental problems?

• How should policies with regard to these problems be carried out in their opinion?

These questions were put to all participants in the three age specific human biomonitoring campaigns: newborn babies (the mothers), adolescents (14/15 years old) and adults (between 50 and 65 years old).

#### Results

About one third of the adolescents and the mothers and almost half of the adults mention the existence of an environmental problem in their neighbourhood [27-29]. The problems mentioned mostly relate to air pollution and are said to be caused mainly by traffic and companies. Of the respondents mentioning the existence of these environmental problems, 44% of the adolescents, 78% of the mothers and 60% of the adults expect there to be a link between theses environmental problems and health risks.

On the issue of trust in actors involved in risk communication and risk management, overall three groups can be distinguished:

• Highest trust: in general practitioners, scientists, environmental organisations

• Moderate trust: in governmental authorities, the media

• Lowest trust: in polluters, politicians

These results on trust to a large extent match results from the Eurobarometer [30]. With regard to participation of members of the public in environmental policymaking, we detected a 'participation paradox'. On the one hand participation of members of the public in environmental policymaking is said to be important by the vast majority of the respondents. On the other hand only a minority of these respondents is willing to actually participate on an individual basis, the main reason being time constraints.

#### Limitations

Risk perception research by means of questionnaires provides an interesting view of respondents' perceptions, but has its limitations, these being mainly caused by the lack of direct interaction between researchers (or their colleagues) and respondents, and between the individual respondents themselves. Of equal importance is the fact that the research does not incorporate developments happening over time; questionnaire results are static. Risk perception is to a large extent constructed through social interaction and develops over time. Furthermore it can only scratch the surface: hidden driving forces remain obscured.

In terms of a strategy of modern risk communication, risk perception research is of value, although it only really constitutes one piece of the puzzle. In a sense it is also a form of one-way communication: from respondents to researchers, so it does not have the quality of a dialogue or cooperation; nor does it directly affect knowledge production.

#### From experiment to an integral part of human biomonitoring research

One of the aims of the perception research was also to integrate social scientific research directly into the non social scientific research on environment and health. This, it more or less succeeded in doing. The perception questionnaire became an integral part of the Flemish human biomonitoring research, for example for the upcoming human biomonitoring campaigns to be carried out by the Centre of Expertise for Environment and Health in its extended period (2007–2011). This does not mean though that the results of perception research are actually used in the work done by non social scientific staff, indicating that some work still has to be done here. Policy representatives see it rather more as information they can act upon, for example with regard to awareness raising campaigns.

### Involvement of local stakeholders

In the second and third year of the Centre of Expertise for Environment and Health we organized two experiments, in which local stakeholders played a central role, in close dialogue with the other researchers and policy makers involved in the work of the Centre. One concerned feedback on aspects of the human biomonitoring research tools, the second related to dialogue on human biomonitoring itself.

#### Reflection on part of the research tools

In this project social scientists designed a relatively simple exercise in order to establish whether cooperation between scientists and local actors on the design of some of the research tools was possible and, if so, useful [31]. Feedback and input was sought on the development of questionnaires to be used in one of the human biomonitoring campaigns and on strategies for the recruitment of participants in the research. This was done via e-mail questionnaires sent out to a variety of local actors in two research areas of the human biomonitoring research.

We invited a number of diverse local actors: residents' groups, companies, labour unions, environmental groups, environmental and health councils, experts and civil servants, and general practitioners. About 25% of the persons we invited participated. Only one type of local actor was not represented: no representatives of environmental organisations participated.

Some of the comments on the questionnaire related to the clarity of the questions. Most of these comments were subsequently taken up in our revision of the questionnaire. Similarly comments were made with regard to privacy: some questions in the questionnaire touched upon sensitive issues. This feedback was also adopted. Feedback concerning co-variables was partly adopted: of the 25 proposals for extra co-variables five were accepted and ten were considered as good candidates. Concerning the recruitment strategy for participants in the human biomonitoring no one clear-cut preference emerged. Some of the respondents criticized a rather confrontational approach of directly inviting people via telephone or mail; at the same a more indirect approach (for example advertisements) also came in for some criticism. The most positive suggestions mostly involved a combination of elements, for example a call via local authority information channels, direct mail and phone calls.

In addition to feedback relating to the questionnaire and recruitment strategy, some interesting comments were made about the research in general. For example the scientific value of statistical relations was criticized; another example concerned dilemmas with regard to environment and health: breast feeding is healthy for the baby but also contains pollutants from the mother, outdoor running is healthy, but what if you live in a polluted area, and so on.

The respondents were informed about the response of the researchers and received the report resulting from the exercise. In the end we can state that the exercise was considered a success. The feedback from local actors was highly appreciated by the human biomonitoring researchers and the opportunity to give input was welcomed by the respondents. We can qualify the exercise as a form of input from stakeholders that influenced the research approach, and as such co-created part of the knowledge production process. Even though there was no direct dialogue between scientists and stakeholders, the openness that is shown by the Centre of Expertise for Environment and Health with such an approach is very well appreciated by the public, as one respondent put it.

#### Scientists and policy makers discuss human biomonitoring with local stakeholders

In one of the human biomonitoring areas, the Ghent Canal Zone, a harbour area, discussions were organized between representatives from the Centre, representing both science and government, and local actors [32]. The main goal was to discuss the expectations of actors about the upcoming research results and the implications for embedment in policy practice and otherwise. The main goal was to collect information for integrating the expectations several actors may have in the communication strategy once research results are ready and to use them as input for follow up strategies.

This process was organized in close cooperation with a local project instigated by the regional authorities in the Ghent harbour area. In this project local stakeholders and authorities discuss and work together on issues that concern the area, of which environmental issues form an important part. Without their support and without the opportunity to make use of an already existing cooperative and deliberative structure of different local actors, the exercise probably would have been much more demanding for the social scientists.

We gave the people who volunteered as participants a voice in the subjects of discussion. They were able to choose between:

• How is the human biomonitoring research designed? Which choices are made?

• How should we communicate about the results of the research?

• What should be done with the research results?

**Figure 3:**
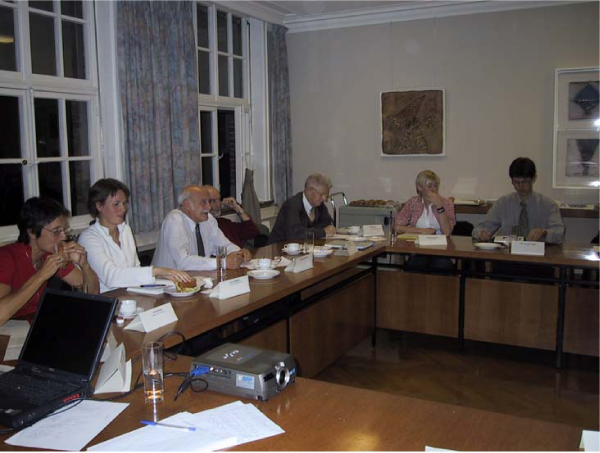
A discussion with local stakeholders on human biomonitoring in the Ghent Canal Zone.

The first and third topic were most popular; we therefore organized two discussion groups, one on research design, and one on the use of (at that stage) future human biomonitoring results. Both public and private actors from the region participated (different persons per discussion): representatives from residents' groups, companies, labour unions, environmental groups, environmental and health councils, experts and civil servants, and general practitioners (see figure 3). From the Centre of Expertise for Environment and Health scientists and representatives from policy making also took part in the discussions.

In the discussion on the design of the research, information was provided on the design of the human biomonitoring on which participants were asked to give feedback. The information concerned the biomarkers, the research areas and the different age groups. It was striking to see that hardly any critical comments were made about the design of the study at the start of the discussion. The main issues brought forward concerned, firstly, the fact that people aged between 15 and 50 were not present in the study and, secondly, focusing attention on skin diseases as a possible health effect. Overall, participants were fairly positive and showed quite a lot of faith in the scientists responsible. Some of the participants wondered if they were expert enough to judge the research design, and one striking remark was made with relevance to the communication of the design of the study: '(...) well, the fact that something is being done is a sign that something is bad. (...) people will think: it must be bad here also, if they are doing research. That's why people should be well informed about the research.'

Some participants in the discussion had high expectations about the research: they believed that the uncertainties arising from the risks from different substances would at last be clarified. In the discussion, however, these expectations were challenged by information relating to the complexity of this field of research.

Representatives of the industry stressed that it was time to look beyond companies as being the only sources of pollution: over the last few decades a lot of improvements have been made by these companies. On the other hand, burning waste in the backyard was presented as one example of individual household pollution which on a collective level may have a serious impact on the environment.

Some participants in the discussion wondered about the usefulness of a discussion on research design, when the research is already underway and choices have already been made. From the side of policy representatives working with the Centre, two reasons were given why it is still useful. 1. It is important to know the type of questions that are raised by the research, so researchers can be prepared. 2. Lessons may be drawn for example for future research activities. Furthermore it was stated that for scientists it is not very common to involve 'others' in the design of their research. More openness though is highly appreciated as was noted during the discussion.

In the discussion group on research results, we used a scenario of an imaginary result to initiate proceedings, since at that stage no real results were available:

Suppose in the Ghent Canal Zone a drastic rise in DNA-damage is detected, without a clear view on the cause. This is only found in this region, and not in other regions. Suppose also that in the Ghent Canal Zone a typical type of industry is present, that is not present in other areas.

The scenario was criticised in the discussion for several reasons. 1. Company representatives stressed that no causal relation was proven with regard to the industry; many other factors may play a role. 2. The question was raised as to whether such a precisely localized problem could realistically be expected from the human biomonitoring, since the relationship between environment and health is very complex involving a host of unknown factors. Human biomonitoring may complete parts of the puzzle, but not necessarily all of it. The type of result sketched out in the scenario was thus not, according to the participants in the discussion, to be expected from the human biomonitoring research. This incidentally was not the original intention of the scenario; it was merely designed to stir up discussions – which it did.

A lot of discussion concerned the difficulties in interpreting the (at that point potential) research results. How should scientifically valid meaning be attributed to the results when so little was known scientifically? And what does this mean for the goal of transparency about research results? A lot of discussion was raised on the question of the 'right' interpretation of results and depending on the background different participants raised different worries and needs. It was pointed out that there exists the danger that non-experts will come up with their own interpretations if science does not have sufficient or unambiguous knowledge to produce clear conclusions. At the same time suspicion was raised with regard to scientific statistical interpretation. As one participant stated, 'you can prove anything with statistics'.

It was stressed that being transparent should also involve information on:

• The aim and design of the research

• The state-of-the-art scientific knowledge, including uncertainties, unknowns and controversies

The issue of transparency is thus given priority over the risk that ambiguous scientific interpretation that may lead to different interpretations by non-scientific actors. With regard to the use of results for policymaking it was stressed that involvement of (local) stakeholder groups is important.

We can qualify the exercise as a form of dialogue between stakeholders and those responsible for both research and policy uptake. Both groups were satisfied with the discussions: researchers and policy makers learned about expectations and views in society, stakeholders learned about the ins and outs of policy relevant scientific research. The outcomes are of value not only for the image of the Centre, but also give inspiration for both risk communication and the policy interpretation of research results.

### Action-plan: interpreting results for policy making

Together with medical and environmental scientific experts and policy makers, social scientists worked on the preparation of an action-plan for the interpretation and policy measures with regard to the human biomonitoring results [33,34]. In the beginning the discussions in the working group mainly focussed on environmental and medical scientific interpretation of the monitoring data. Consultation of scientific experts as well as desk research was considered to provide the necessary knowledge and answers. Later on in the conceptual process other elements were introduced by the social scientists: complementary assessment criteria, complementary assessment methods and involvement of other actors in the process.

In three successive analytical phases, the human biomonitoring results are assessed on different aspects. The first phase focuses on the question: how severe are specific results with regard to public health risks? To a large extent in this phase the discussion focuses on reference values for interpreting the data. This is quite problematic since knowledge of these issues is still rather limited. Only with regard to lead (international) norms are available. Therefore an average reference value per pollutant or health effect is used to decide which human biomonitoring results are relatively high. A comparison is also done with research outcomes from other studies e.g. from abroad. The second phase focuses on the question: what are the causes for a specific monitoring result? For example, causes may be environmentally related or life style related. In the third and final phase the focus is on the question: can we identify a (local) source for the pollution?

At first the action-plan was thought of as a merely scientific quest: with the right group of experts the interpretation with regard to policy priorities will follow automatically. While trying to build bridges towards policy interpretation though, the limitations of an exclusively scientific endeavour were clearly evident: no scientist or group of scientists dared to claim that they possessed the necessary and overarching knowledge for answering difficult questions – questions e.g. on policy priorities when factors other than (medical and environmental) scientific ones also had to be taken into account (economics, social preferences, feasibility of policy measures; issues introduced by the social scientists). The social scientists therefore proposed the formation of a jury that will judge relevant data and knowledge in order to give advice to the government.

**Figure 4:**
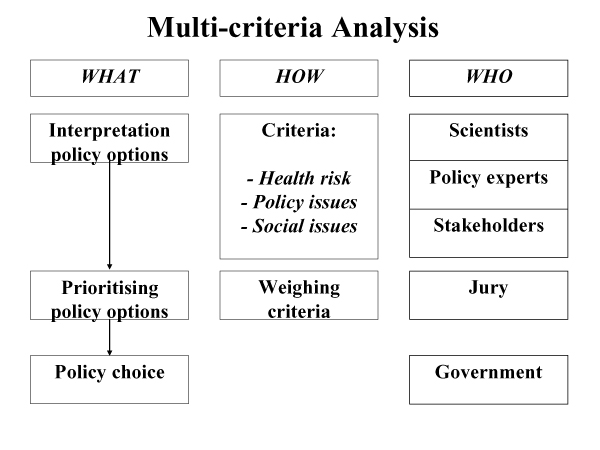
Multi-criteria Analysis.

Furthermore the need for a stepwise procedure 'from data-interpretation to decision making' became an urgent priority. In order to achieve this, the social scientists developed a practice cycle with the different procedural steps, actors and roles for each phase: from assessment to decision making. The practice cycle is made up of cyclical steps to be taken during each phase of the action-plan: deciding how to operate and which actors to involve during the process, desk research on the human biomonitoring results and expert consultation, bringing a synthesis of the desk research and expert consultation before a jury of stakeholders, synthesis of desk research, expert consultation and jury advice for the administration. In the end it is the government that decides the next steps. During all of these steps, external communication about the process is included. For the expert round we use an e-mail questionnaire. For the jury we use a multi-criteria method (figure 4) and a group discussion.

In 2006 a first pilot project was carried out to test part of the conceptual action-plan: the second phase of the action-plan was tested on the DDE-results of the human biomonitoring [33]. In 2007 another pilot was started looking at the prioritization of results for policy making (phase one of the action-plan). Although it is too early to draw definitive conclusions about the conceptual action-plan at this stage, one thing is clear; this is not an 'exercise behind closed doors' or a low profile endeavour. The ministers of Environment and Health have publicly mentioned the action-plan on several occasions as the instrument for translating the human biomonitoring research results to policy-making.

## Discussion

We will briefly discuss risk communication with regard to human biomonitoring in the European Union (EU). Between March 11th–14th 2007, in Copenhagen (Denmark) an ESBIO (Expert team to Support human BIOmonitoring in Europe – an EU-funded project) [35] workshop was held on ethics and communication. In this workshop the risk communication activities described here were presented and discussed.

Within the EU, practices with respect to communication on human biomonitoring are differing widely, and there is general lack of expert reflection on modern risk communication. The EU human biomonitoring pilot study has aims to harmonise practices at EU level and would profit from an advanced risk communication plan. However, without the actual presence of social scientific experts with relevant experience in the core of their activities, a reflex of modern risk communication may not develop, despite the EU views on risk communication, transparency and participation of which the approval of the Aarhus Convention [16] is but one good example.

Similar to the strategy within the Flemish biomonitoring, traditional communication from the start could run in parallel with a step by step internal reflection on and experiments with modern risk communication. At the same time though we must be aware that the stage is different here, since the constellation of an EU-project is different from the one in a single country. The international perspective including different countries with different cultures and histories, different policies and discourse on environment and health, different relations between science, policy and society, different experiences with risk communication, etc. implies a need to look at the risk communication issue both from an EU and a national perspective.

At the EU-level more general concepts and strategies may be discussed and developed, but these must to some extent be flexible in order to 'fit' different national settings, according to the subsidiary principle. The risk communication activities at the national level preferably need to be addressed by local experts with experience in the field of risk communication. Risk communication concepts and methods developed on EU-level, need to be adapted to the local settings to be effective. Feedback loops should be developed in order to organize exchange of experiences and good practices between countries and in order to reflect the overall activities at a more general level. In an interactive and iterative process of reflection and learning, step by step modern risk communication activities can thus be developed in close cooperation with all actors involved and adapted to both the general EU-level and the national contexts.

## Conclusion

We have sketched how within the framework of the Flemish Centre of Expertise for Environment and Health a parallel strategy was developed of traditional risk communication on the one hand and internal reflection on and experiments with modern risk communication on the other. Step by step in a process characterized by cooperation and learning by doing, we tried to adapt the ideal theoretical concepts of the 'Ten Commandments level' to both day-to-day practice and to the level of relevant experience and knowledge of our colleagues and their preferences. Thus, over time, the traditional one-way communication activities from experts to the public were supplemented with modern risk communication experiments, of which some developed to mature integral parts of the work of the Centre of Expertise for Environment and Health.

As a counterpart to one-way communication from expert to the public, a risk perception questionnaire was developed to monitor not only pollutants and health effects, but also people's perceptions of environment and health issues. Another form of communication from the public to the experts was the feedback on some of the research tools: a questionnaire and recruitment strategy. A more dialogical experiment was organized with discussions between representatives from the Centre and local stakeholders on the design of the research and interpretation of research results. These (and other) experiments and projects helped, we believe, pave the way for the social scientific contribution to for example the action-plan for the interpretation of research results. The general research question of how to incorporate principles from modern risk communication into the day-to-day practice of human biomonitoring research is therefore answered both by the parallel strategy of traditional and modern communication, of external and internal reflection, and by the different social scientific projects over the past 5 years. In this respect, concepts and methods related to modern risk communication were able to crystallize and grow to integral parts of the interdisciplinary (different scientific disciplines) and transdisciplinary (involvement of non-scientific actors, here mainly policy makers) endeavour.

Important success factors are the direct and continuous involvement of social scientists as well as the openness of colleagues from other scientific disciplines as well as from policymaking. Another success factor is the combined strategy as described above. Also of importance is the awareness of a fine balance between quality and practicality. This needs continuous attention and reflection with the actors involved.

With regard to the EU-perspective of risk communication and human biomonitoring these lessons may be of help. We believe that the complexity and expertise of good quality modern risk communication is vastly underestimated with regard to risk communication on human biomonitoring in the EU. Non-social scientific experts showed this reflex of underestimation also in the beginning of the Centre of Expertise for Environment and Health in Flanders. It takes time and joint effort to make this work. Within the EU-context the need for a subsidiary approach from the general EU-level to the different national context will be an extra complicating factor. The Flemish experience will hopefully be of inspiration for the EU endeavour and has proven to be very fruitful during five years of interesting practice.

## Competing interests

The authors declare that they have no competing interests.

## Authors' contributions

HK carried out most of the research and field work during the past five years, IL participated in this effort as a supervisor. HK is responsible for the description, analysis and interpretation of the fieldwork as well as the evaluation with regard to the EU-perspective. IL had a reflective role in this respect. BM joined the work more recently. BM helped in editing the text and did some supplementary literature research with regard to risk communication and methodology.
